# Supplementary material for: Global Quantitative Analysis of Ligation Reactions in Self‐Assembled DNA Nanostructures at the Single‐Nick Level
Source: Small. 2026 Apr 29;22(33):e08136. doi: 10.1002/smll.202508136 (PMC13262551; doi:10.1002/smll.202508136)
Supplement: Supplementary file 1 — Supporting File: smll73444‐sup‐0001‐SuppMat.pdf. [file SMLL-22-e08136-s001.pdf]

Supplementary information for

Global Quantitative Analysis of Ligation Reactions in Self-Assembled DNA Nanostructures at the Single-Nick Level

Konrad Hacker [1,4], Emilia Juricke [2,4], Carolin Münch [1], Antonio Suma [3,]*, Adrian Keller [2,]*, Yixin Zhang [1,5]*

1. B CUBE — Center for Molecular Bioengineering, Technische Universität Dresden, Dresden (Germany)
2. Technical and Macromolecular Chemistry, Paderborn University, Paderborn (Germany)
3. Dipartimento Interateneo di Fisica, Università degli Studi di Bari and INFN, Sezione di Bari, Bari (Italy)
4. These authors contribute equally to this work.

* For correspondence: A.S. (antonio.suma@uniba.it); A.K. (adrian.keller@uni-paderborn.de); Y.Z. (yixin.zhang1@tu-dresden.de)

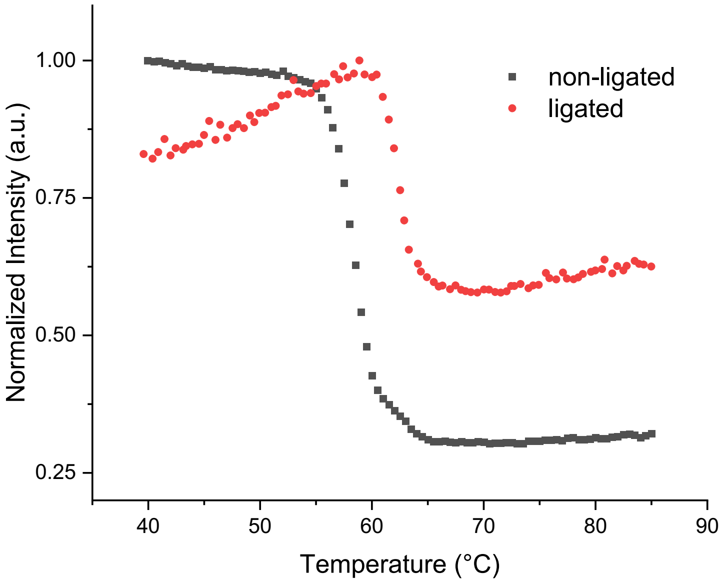

Figure S1. Normalized scattering intensity of ligated and non-ligated DON in water over a temperature range from 40°C to 85°C.

Table 1. *General thermal cycler program for quantification using qPCR*. Annealing temperature is dependent on the specific primer pair melting temperature*.

steps	scan	temperature (°C)	time (m:s)	go to	loops	temperature increments (°C/s)
1		95	01:00			8
2		95	00:10			6
3		~50*	00:05			6
4	♦	72	00:05	2	40	6
5		60	00:05			6
6		60 to 95	08:45			$^{1}/_{15}$

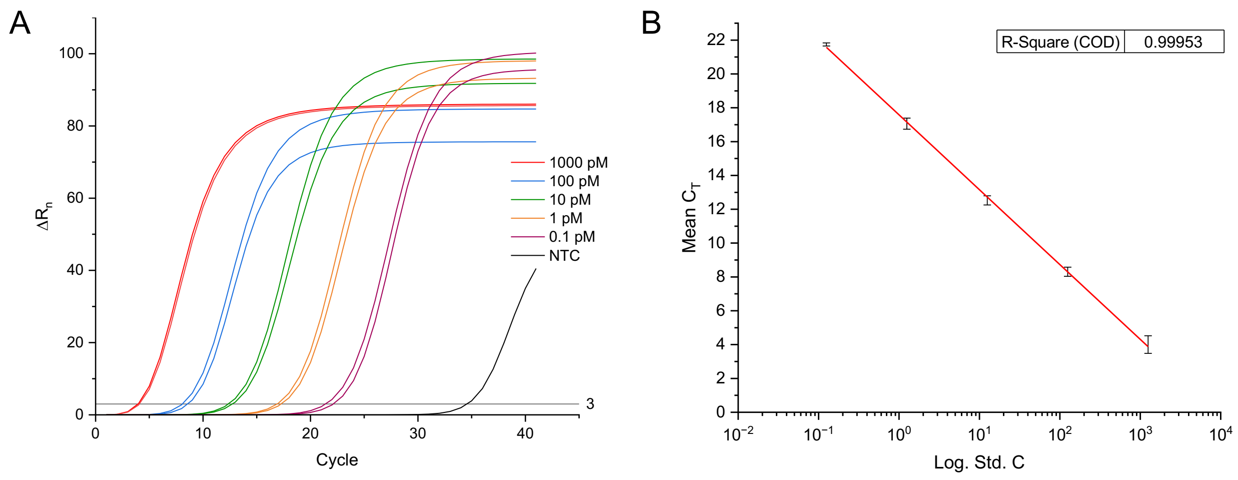

Figure S2. (A) Amplification curves of a selected sequence from the scaffold in tenfold dilutions and negative control (NTC). (B) Calibration curve of the selected sequence.

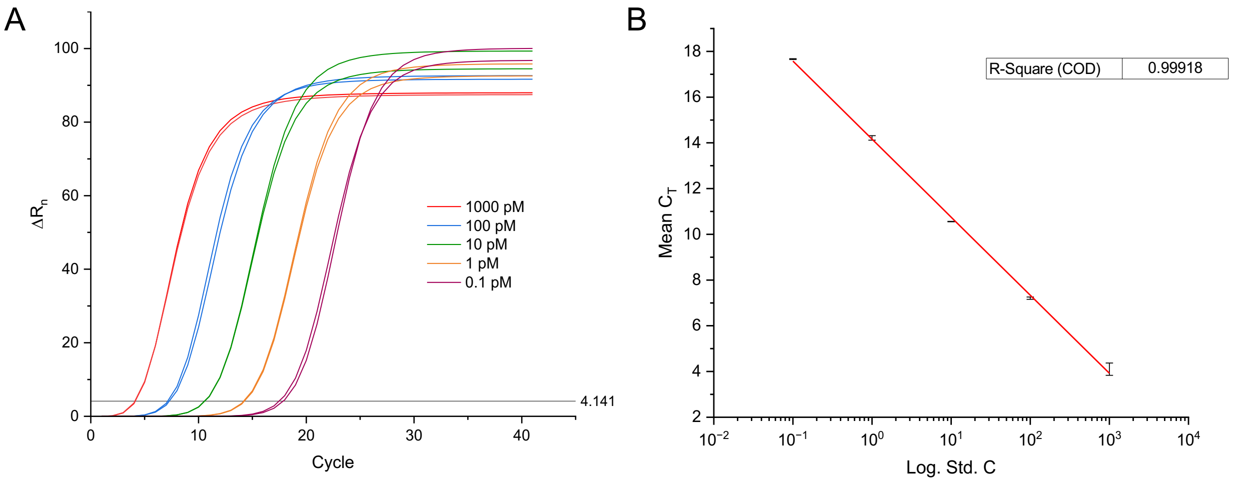

Figure S3. (A) Amplification curves of the sequence corresponding to the ligation product of the pair of staple strands at nick 1 in tenfold dilutions. (B) Calibration curve of the DNA sequence.

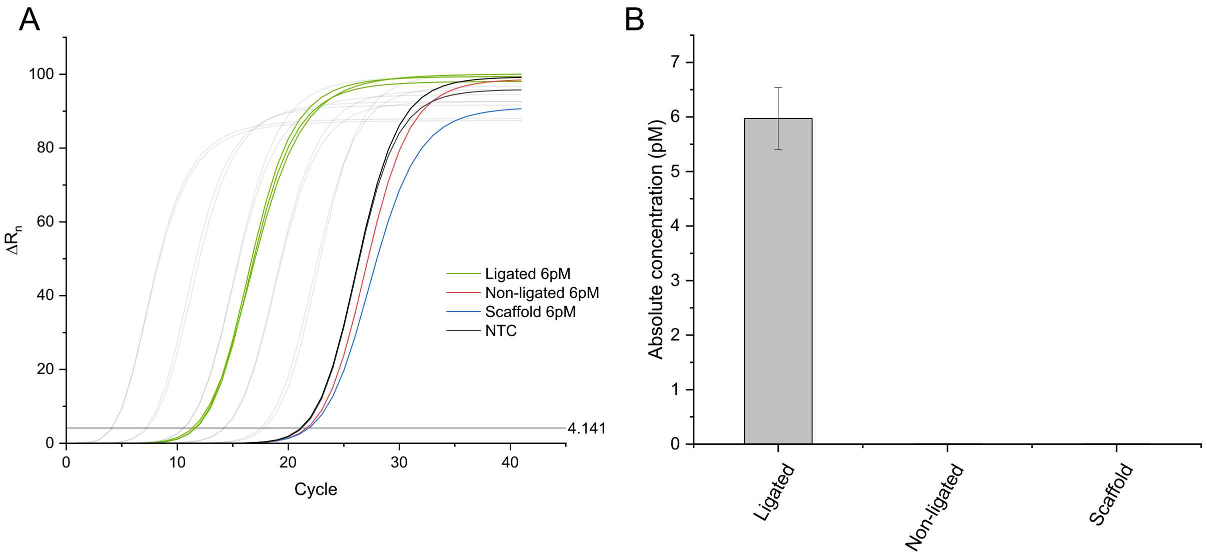

Figure S4. S*pecific amplification and quantification of the ligation product at nick 1 on DON, but not the staple strands and scaffold.* (A) Amplification curves of the ligated and non-ligated DON samples as well as the scaffold sample. (B) Calculated concentrations of the sequence with ligated, non-ligated and scaffold sample measured by qPCR and calculated according to the calibration curve in figure **S2B**.

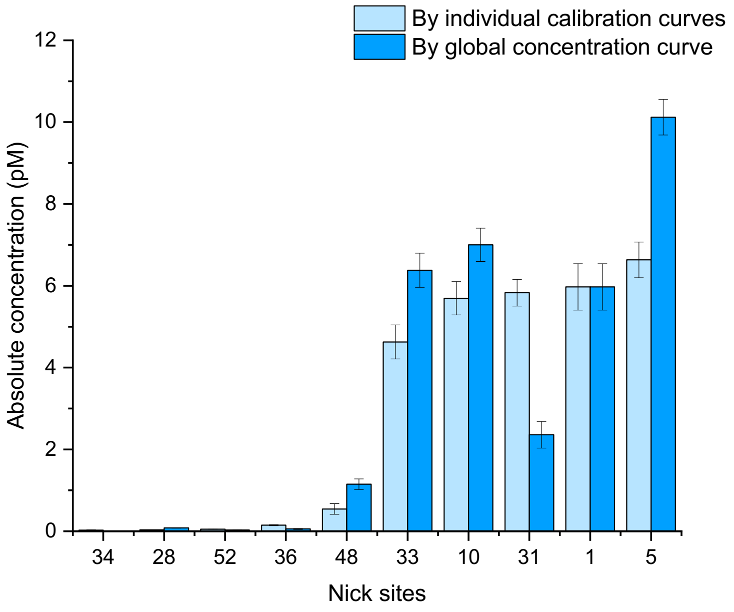

Figure S5. Comparison of the calculated concentrations derived from individual calibration curves or from the global calibration curve at nick 1 (figure **S2B**) for ten staple strand pairs.

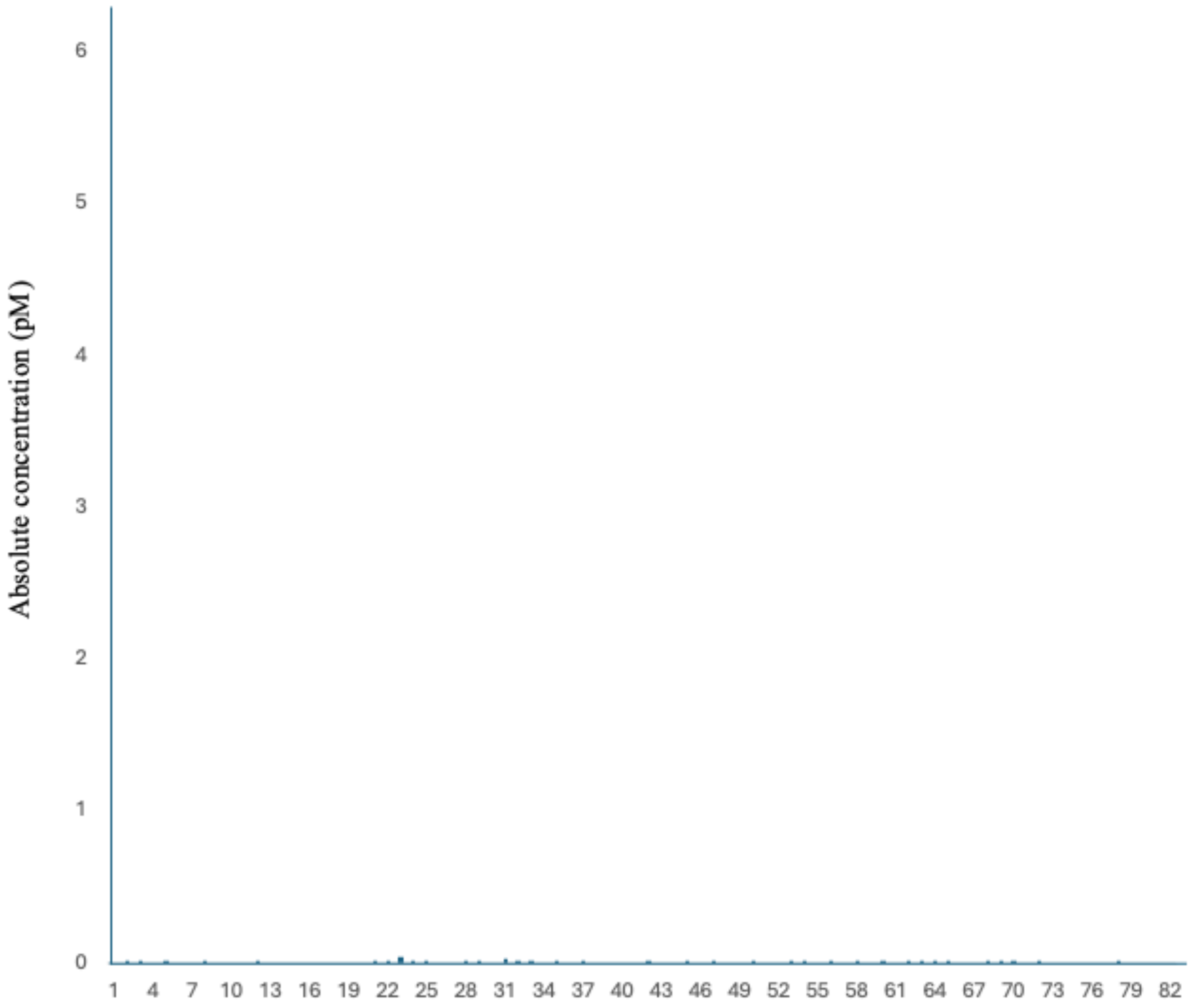

Figure S6. Shot gun analyses with 82 random pairs of primers**.**

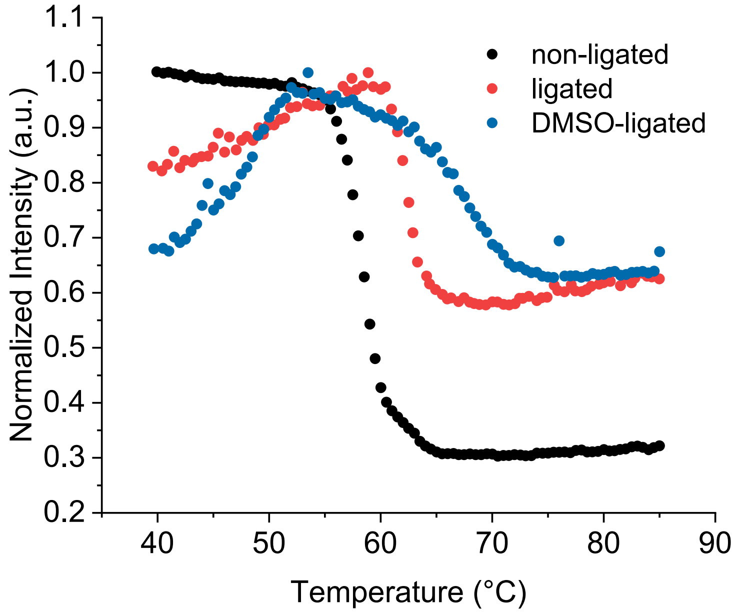

Figure S7. Temperature dependence of scattering intensity of triangular DNA origami structures without ligation, with ligation by T4 DNA ligase and with enzymatic ligation in the presence of DMSO.

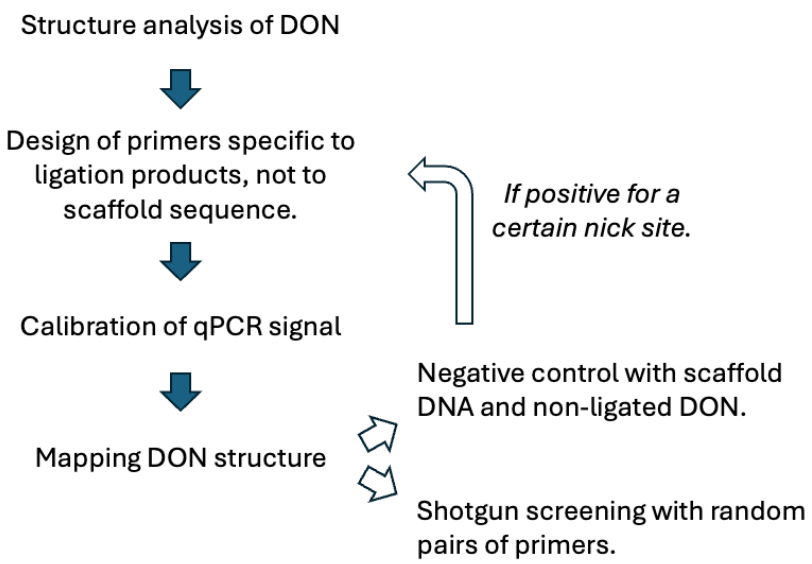

Scheme S1. Global quantitative analysis of ligation reactions work flow.

Table S2. *Staple strand pairs for ligation.*

Nick #	Staple 1	Sequence	Staple 2	Sequence	Total sequence	Total length	Primer 1	T_m (°C)	Primer 2	T_m (°C)	T_{anneal} (°C)
1	t-10s7h	ACGACAATAAATCCCGACTTGCGGGAGATCCTGAATCTTACCA	t-9s10g	ACGCTAACGAGCGTCTGGCGTTTTAGCGAACCCAACATGT	ACGACAATAAATCCCGACTTGCGGGAGATCCTGAATCTTACCAACGCTAACGAGCGTCTGGCGTTTTAGCGAACCCAACATGT	83	ACGACAATAAATCCCGACTT	52	ACATGTTGGGTTCGCTAAAA	53	49
2	t-7s8g	GCGCCTGTTATTCTAAGAACGCGATTCCAGAGCCTAATTT	t-7s10g	GCCAGTTACAAAATAATAGAAGGCTTATCCGGTTATCAAC	GCGCCTGTTATTCTAAGAACGCGATTCCAGAGCCTAATTTGCCAGTTACAAAATAATAGAAGGCTTATCCGGTTATCAAC	80	GCGCCTGTTATTCTAAGAAC	53	GTTGATAACCGGATAAGCCT	53	49
3	t-5s8g	ACAAGAAAGCAAGCAAATCAGATAACAGCCATATTATTTA	t-5s10g	TCCCAATCCAAATAAGATTACCGCGCCCAATAAATAATAT	ACAAGAAAGCAAGCAAATCAGATAACAGCCATATTATTTATCCCAATCCAAATAAGATTACCGCGCCCAATAAATAATAT	72	ACAAGAAAGCAAGCAAATCA	57	ATATTATTTATTGGGCGCGG	51	49
4	t-3s8g	AGCATGTATTTCATCGTAGGAATCAAACGATTTTTTGTTT	t-3s10g	AACGTCAAAAATGAAAAGCAAGCCGTTTTTATGAAACCAA	AGCATGTATTTCATCGTAGGAATCAAACGATTTTTTGTTTAACGTCAAAAATGAAAAGCAAGCCGTTTTTATGAAACCAA	80	AGCATGTATTTCATCGTAGGAATCA	51	TTGGTTTCATAAAAACGGCTTGCTT	51	49
5	t-1s8g	TTTCCTTAGCACTCATCGAGAACAATAGCAGCCTTTACAG	t-1s10e	AGAGAATAACATAAAAACAGGGAAGCGCATTA	TTTCCTTAGCACTCATCGAGAACAATAGCAGCCTTTACAGAGAGAATAACATAAAAACAGGGAAGCGCATTA	72	TTTCCTTAGCACTCATCGAGAACA	57	TAATGCGCTTCCCTGTTTTT	53	50
6	t-1s10e	AGAGAATAACATAAAAACAGGGAAGCGCATTA	t1s10g	GACGGGAGAATTAACTCGGAATAAGTTTATTTCCAGCGCC	AGAGAATAACATAAAAACAGGGAAGCGCATTAGACGGGAGAATTAACTCGGAATAAGTTTATTTCCAGCGCC	72	AGAGAATAACATAAAAACAGGGAAG	51	GGCGCTGGAAATAAACTTAT	52	49
7	t3s8g	CATTCAACAAACGCAAAGACACCAGAACACCCTGAACAAA	t3s10g	GTCAGAGGGTAATTGATGGCAACATATAAAAGCGATTGAG	CATTCAACAAACGCAAAGACACCAGAACACCCTGAACAAAGTCAGAGGGTAATTGATGGCAACATATAAAAGCGATTGAG	80	CATTCAACAAACGCAAAGAC	52	CTCAATCGCTTTTATATGTTGC	51	49
8	t5s8g	TTGACGGAAATACATACATAAAGGGCGCTAATATCAGAGA	t5s10g	GATAACCCACAAGAATGTTAGCAAACGTAGAAAATTATTC	TTGACGGAAATACATACATAAAGGGCGCTAATATCAGAGAGATAACCCACAAGAATGTTAGCAAACGTAGAAAATTATTC	80	TTGACGGAAATACATACATAAAG	49	GAATAATTTTCTACGTTTGCTAAC	49	49
9	t7s8g	CACCGTCACCTTATTACGCAGTATTGAGTTAAGCCCAATA	t7s10g	ATAAGAGCAAGAAACATGGCATGATTAAGACTCCGACTTG	CACCGTCACCTTATTACGCAGTATTGAGTTAAGCCCAATAATAAGAGCAAGAAACATGGCATGATTAAGACTCCGACTTG	80	CACCGTCACCTTATTACGCA	57	CAAGTCGGAGTCTTAATCAT	50	49
10	t9s8g	GAGCCAGCGAATACCCAAAAGAACATGAAATAGCAATAGC	t9s10h	TATCTTACCGAAGCCCAAACGCAATAATAACGAAAATCACCAG	GAGCCAGCGAATACCCAAAAGAACATGAAATAGCAATAGCTATCTTACCGAAGCCCAAACGCAATAATAACGAAAATCACCAG	83	GAGCCAGCGAATACCCAAAA	57	CTGGTGATTTTCGTTATTATTGC	51	48

Nick #	Staple 1	Sequence	Staple 2	Sequence	Total sequence	Total length	Primer 1	T_m (°C)	Primer 2	T_m (°C)	T_{anneal} (°C)
11	t-12s9h	TGCTATTTTGCACCCA GCTACAATTTTGTTTTG AAGCCTTAAA	t-11s8e-t12s29c-0T	TCAAGATTAGTGTAGCA ATACT	TGCTATTTTGCACCCAGCTACAATT TTGTTTTGAAGCCTTAAATCAAGAT TAGTGTAGCAATACT	65	TGCTATTTTGC ACCCAGCTA	56	AGTATTGCT ACACTAATC TTGA	49	46
12	t1s8i	ATGGTTTATGTCACAAT CAATAGATATTAAAC	t-1s8i	CAAGTACCTCATTCCAA GAACGGGAAATTCAT	ATGGTTTATGTCACAATCAATAGAT ATTAAACCAAGTACCTCATTCCAAG AACGGGAAATTCAT	64	ATGGTTTATGTC ACAATCAATAG AT	51	ATGAATTTC CCGTTCTTG GA	53	49
13	t-11s18e-t12s9c-0T	ATAAGGCTTGCAACAA AGTTAC	t11s8h	CAGAAGGAAACCGAGG TTTTTAAGAAAAGTAA GCAGATAGCCG	ATAAGGCTTGCAACAAAGTTACCA GAAGGAAACCGAGGTTTTTAAGAA AAGTAAGCAGATAGCCG	65	ATAAGGCTTGC AACAAAGTT	51	CGGCTATCT GCTTACTTTT C	52	49
14	t-9s10g	ACGCTAACGAGCGTCT GGCGTTTTAGCGAACC CAACATGT	t-8s7c	TCAGCTAAAAAAGGTA AAGTAATT	ACGCTAACGAGCGTCTGGCGTTTT AGCGAACCCAACATGTTCAGCTAA AAAAGGTAAAGTAATT	64	ACGCTAACGAG CGTCTGGCG	65	AATTACTTTA CCTTTTTTAG CTGA	48	46
15	t-8s5f	TTCTGACCTAAAATATA AAGTACCGACTGCAGA AC	t-7s8g	GCGCCTGTTATTCTAAG AACGCGATTCCAGAGC CTAATTT	TTCTGACCTAAAATATAAAGTACCG ACTGCAGAACGCGCCTGTTATTCTA AGAACGCGATTCCAGAGCCTAATT T	75	TTCTGACCTAA AATATAAAGTA CCG	51	AAATTAGGC TCTGGAATC GC	54	49
16	t-7s10g	GCCAGTTACAAAATAA TAGAAGGCTTATCCGG TTATCAAC	t-6s7f	AATAGATAGAGCCAGTA ATAAGAGATTTAATG	GCCAGTTACAAAATAATAGAAGGC TTATCCGGTTATCAACAATAGATAG AGCCAGTAATAAGAGATTTAATG	80	GCCAGTTACAA AATAATAGAAG G	53	CATTAAATCT CTTATTACTG GCT	53	49
17	t-5s6e	GTGTGATAAGGCAGAG GCATTTTCAGTCCTGA	t-5s8g	ACAAGAAAGCAAGCAA ATCAGATAACAGCCATA TTATTTA	GTGTGATAAGGCAGAGGCATTTTC AGTCCTGAACAAGAAAGCAAGCA AATCAGATAACAGCCATATTATTTA	67	GTGTGATAAGG CAGAGGCAT	54	TAAATAATAT GGCTGTTAT CTGATT	55	49
18	t-5s10g	TCCCAATCCAAATAAG ATTACCGCGCCCAATA AATAATAT	t-4s7f	CCCATCCTCGCCAACAT GTAATTTAATAAGGC	TCCCAATCCAAATAAGATTACCGCG CCCAATAAATAATATCCCATCCTCG CCAACATGTAATTTAATAAGGC	80	TCCCAATCCAA ATAAGATTACC	51	GCCTTATTAA ATTACATGTT GGC	52	49
19	t-3s6e	CACCGGAATCGCCATA TTTAACAAAATTTACG	t-3s8g	AGCATGTATTTCATCGT AGGAATCAAACGATTTT TTGTTT	CACCGGAATCGCCATATTTAACAAA ATTTACGAGCATGTATTTCATCGTA GGAATCAAACGATTTTTTGTTT	72	CACCGGAATCG CCATATTTAACA AA	54	AAACAAAA AATCGTTTG ATTCCTA	50	49
20	t-3s10g	AACGTCAAAAATGAA AAGCAAGCCGTTTTTA TGAAACCAA	t-2s7f	TCAATAATAGGGCTTAA TTGAGAATCATAATT	AACGTCAAAAATGAAAAGCAAGC CGTTTTTATGAAACCAATCAATAAT AGGGCTTAATTGAGAATCATAATT	72	AACGTCAAAA ATGAAAAGCAA	50	AATTATGATT CTCAATTAA GCCC	49	46

Nick #	Staple 1	Sequence	Staple 2	Sequence	Total sequence	Total length	Primer 1	T_m (°C)	Primer 2	T_m (°C)	T_{anneal} (°C)
21	t-1s6e	TTAGTATCGCCAACGCTCAACAGTCGGCTGTC	t-1s8g	TTTCCTTAGCACTCATCGAGAACAATAGCAGCCTTTACAG	TTAGTATCGCCAACGCTCAACAGTCGGCTGTCTTTCCTTAGCACTCATCGAGAACAATAGCAGCCTTTACAG	72	TTAGTATCGCCAACGCTCAA	55	CTGTAAAGGCTGCTATTGTT	51	49
22	t-1s8i	CAAGTACCTCATTCCAAGAACGGGAAATTCAT	t1s8i	ATGGTTTATGTCACAATCAATAGATATTAAAC	CAAGTACCTCATTCCAAGAACGGGAAATTCATATGGTTTATGTCACAATCAATAGATATTAAAC	64	CAAGTACCTCATTCCAAGAA	50	GTTTAATATCTATTGATTGTGACAT	47	46
23	t1s10g	GACGGGAGAATTAACTCGGAATAAGTTTATTTCCAGCGCC	t2s7f	AAAGACAACATTTTCGGTCATAGCCAAAATCA	GACGGGAGAATTAACTCGGAATAAGTTTATTTCCAGCGCCAAAGACAACATTTTCGGTCATAGCCAAAATCA	72	GACGGGAGAATTAACTCGGA	56	TGATTTTGGCTATGACCGAA	53	49
24	t3s6e	CACCGGAAAGCGCGTTTTCATCGGAAGGGCGA	t3s8g	CATTCAACAAACGCAAAGACACCAGAACACCCTGAACAAA	CACCGGAAAGCGCGTTTTCATCGGAAGGGCGACATTCAACAAACGCAAAGACACCAGAACACCCTGAACAAA	72	CACCGGAAAGCGCGTTTTCA	61	TTTGTTCAGGGTGTTCTGGT	56	54
25	t3s10g	GTCAGAGGGTAATTGATGGCAACATATAAAAGCGATTGAG	t4s7f	GGAGGGAATTTAGCGTCAGACTGTCCGCCTCC	GTCAGAGGGTAATTGATGGCAACATATAAAAGCGATTGAGGGAGGGAATTTAGCGTCAGACTGTCCGCCTCC	72	GTCAGAGGGTAATTGATGGC	55	GGAGGCGGACAGTCTGACGC	66	54
26	t5s6e	TCAGAACCCAGAATCAAGTTTGCCGGTAAATA	t5s8g	TTGACGGAAATACATACATAAAGGGCGCTAATATCAGAGA	TCAGAACCCAGAATCAAGTTTGCCGGTAAATATTGACGGAAATACATACATAAAGGGCGCTAATATCAGAGA	72	TCAGAACCCAGAATCAAGTT	52	TCTCTGATATTAGCGCCCTT	54	49
27	t5s10g	GATAACCCACAAGAATGTTAGCAAACGTAGAAAATTATTC	t6s7f	ATTAAAGGCCGTAATCAGTAGCGAGCCACCCT	GATAACCCACAAGAATGTTAGCAAACGTAGAAAATTATTCATTAAAGGCCGTAATCAGTAGCGAGCCACCCT	72	GATAACCCACAAGAATGTTAGC	53	AGGGTGGCTCGCTACTGATT	61	49
28	t7s6e	AGAGCCGCACCATCGATAGCAGCATGAATTAT	t7s8g	CACCGTCACCTTATTACGCAGTATTGAGTTAAGCCCAATA	AGAGCCGCACCATCGATAGCAGCATGAATTATCACCGTCACCTTATTACGCAGTATTGAGTTAAGCCCAATA	72	AGAGCCGCACCATCGATAGC	62	TATTGGGCTTAACTCAATACTGC	54	49
29	t7s10g	ATAAGAGCAAGAAACATGGCATGATTAAGACTCCGACTTG	t8s7g	AGCCATTTAAACGTCACCAATGAACACCAGAACCA	ATAAGAGCAAGAAACATGGCATGATTAAGACTCCGACTTGAGCCATTTAAACGTCACCAATGAACACCAGAACCA	75	ATAAGAGCAAGAAACATGGC	52	TGGTTCTGGTGTTCATTGGT	56	49
30	t9s6e	CCATTAGCAAGGCCGGGGGAATTA	t9s8g	GAGCCAGCGAATACCCAAAAGAACATGAAATAGCAATAGC	CCATTAGCAAGGCCGGGGGAATTAGAGCCAGCGAATACCCAAAAGAACATGAAATAGCAATAGC	64	CCATTAGCAAGGCCGGGGGA	65	GCTATTGCTATTTCATGTTCTTT	50	49

Nick #	Staple 1	Sequence	Staple 2	Sequence	Total sequence	Total length	Primer 1	T_m (°C)	Primer 2	T_m (°C)	T_{anneal} (°C)
31	t-8s7c	TCAGCTAAAAAAGGTA AAGTAATT	t-9s6e-t10s27c-1T	TCAGCTAAAAAAGGT AAAGTAATT	TCAGCTAAAAAAGGTAAAGTAATT CTGTCCAGACGTATACCGAACGA	47	TCAGCTAAAAA AGGTAAAGTAA TT	48	TCGTTCGGT ATACGTCTG GA	56	46
32	t1s6i	TTCATAATCCCCTTATT AGCGTTTTTCTTACC	t-1s6i	AGTATAAAATATGCGT TATACAAAGCCATCTT	TTCATAATCCCCTTATTAGCGTTTTT CTTACCAGTATAAAATATGCGTTATA CAAAGCCATCTT	64	TTCATAATCCCC TTATTAGCG	50	AAGATGGCT TTGTATAAC GC	52	49
33	t-9s16e-t10s7c-1T	ACTACGAAGGCTTAGC ACCATTA	t9s6e	ACTACGAAGGCTTAG CACCATTA	ACTACGAAGGCTTAGCACCATTAC CATTAGCAAGGCCGGGGGAATTA	47	ACTACGAAGGC TTAGCACCA	57	TAATTCCCCC GGCCTTGCT A	60	54
34	t-6s7f	AATAGATAGAGCCAGT AATAAGAGATTTAATG	t-6s5c	GTTTGAAATTCAAAT ATATTTTAG	AATAGATAGAGCCAGTAATAAGAG ATTTAATGGTTTGAAATTCAAATAT ATTTTAG	56	AATAGATAGAG CCAGTAATA	44	CTAAAATATA TTTGAATTTC AAAC	43	40
35	t-6s3f	TCCCTTAGAATAACGC GAGAAAACTTTTACCG ACC	t-5s6e	GTGTGATAAGGCAGA GGCATTTTCAGTCCT GA	TCCCTTAGAATAACGCGAGAAAAC TTTTACCGACCGTGTGATAAGGCA GAGGCATTTTCAGTCCTGA	72	TCCCTTAGAAT AACGCGAGA	50	TCAGGACTG AAAATGCCT CT	48	46
36	t-4s7f	CCCATCCTCGCCAACA TGTAATTTAATAAGGC	t-4s5f	GTTAAATACAATCGC AAGACAAAGCCTTGA AA	CCCATCCTCGCCAACATGTAATTTA ATAAGGCGTTAAATACAATCGCAA GACAAAGCCTTGAAA	64	CCCATCCTCGC CAACATGTA	59	TTTCAAGGC TTTGTCTTG CG	55	52
37	t-3s4e	GATTAAGAAATGCTGA TGCAAATCAGAATAAA	t-3s6e	CACCGGAATCGCCAT ATTTAACAAAATTTAC G	GATTAAGAAATGCTGATGCAAATC AGAATAAACACCGGAATCGCCATAT TTAACAAAATTTACG	64	GATTAAGAAAT GCTGATGCAA	50	CGTAAATTTT GTTAAATATG GCGA	50	49
38	t-2s7f	TCAATAATAGGGCTTA ATTGAGAATCATAATT	t-2s5f	ACTAGAAATATATAAC TATATGTACGCTGAGA	TCAATAATAGGGCTTAATTGAGAAT CATAATTACTAGAAATATATAACTAT ATGTACGCTGAGA	64	TCAATAATAGG GCTTAATTGAG A	49	TCTCAGCGT ACATATAGTT A	48	46
39	t-1s4e	TTATCAAACCGGCTTA GGTTGGGTAAGCCTGT	t-1s6e	TTAGTATCGCCAACG CTCAACAGTCGGCTG TC	TTATCAAACCGGCTTAGGTTGGGTA AGCCTGTTTAGTATCGCCAACGCTC AACAGTCGGCTGTC	64	TTATCAAACCG GCTTAGGTT	53	GACAGCCGA CTGTTGAGC GT	63	49
40	t-1s6i	AGTATAAAATATGCGTT ATACAAAGCCATCTT	t1s6i	TTCATAATCCCCTTAT TAGCGTTTTTCTTACC	AGTATAAAATATGCGTTATACAAAG CCATCTTTTCATAATCCCCTTATTAG CGTTTTTCTTACC	64	AGTATAAAATAT GCGTTATACAA AG	47	GGTAAGAAA AACGCTAAT AAG	47	46

Nick #	Staple 1	Sequence	Staple 2	Sequence	Total sequence	Total length	Primer 1	T_m (°C)	Primer 2	T_m (°C)	T_{anneal} (°C)
41	t2s7f	AAAGACAACATTTTCGGTCATAGCCAAAATCA	t2s5f	CCGGAACCCAGAATGGAAAGCGCAACATGGCT	AAAGACAACATTTTCGGTCATAGCCAAAATCACCGGAACCCAGAATGGAAAGCGCAACATGGCT	64	AAAGACAACATTTTCGGTCA	51	AGCCATGTTGCGCTTTCCAT	60	49
42	**t3s4e**	**TGTACTGGAAATCCTCATTAAAGCAGAGCCAC**	**t3s6e**	**CACCGGAAAGCGCGTTTTCATCGGAAGGGCGA**	**TGTACTGGAAATCCTCATTAAAGCAGAGCCACCACCGGAAAGCGCGTTTTCATCGGAAGGGCGA**	**64**	**TGTACTGGAAATCCTCATTAAA**	**50**	**TCGCCCTTCCGATGAAAACG**	**60**	**49**
43	t4s7f	GGAGGGAATTTAGCGTCAGACTGTCCGCCTCC	t4s5f	CTCAGAGCATATTCACAAACAAATTAATAAGT	GGAGGGAATTTAGCGTCAGACTGTCCGCCTCCCTCAGAGCATATTCACAAACAAATTAATAAGT	64	GGAGGGAATTTAGCGTCAGA	56	ACTTATTAATTTGTTTGTGAATATG	46	41
44	**t5s4e**	**CCTTGAGTCAGACGATTGGCCTTGCGCCACCC**	**t5s6e**	**TCAGAACCCAGAATCAAGTTTGCCGGTAAATA**	**CCTTGAGTCAGACGATTGGCCTTGCGCCACCCTCAGAACCCAGAATCAAGTTTGCCGGTAAATA**	**64**	**CCTTGAGTCAGACGATTGGC**	**58**	**TATTTACCGGCAAACTTGAT**	**50**	**49**
45	**t6s7f**	**ATTAAAGGCCGTAATCAGTAGCGAGCCACCCT**	**t6s5g**	**CAGAGCCAGGAGGTTGAGGCAGGTAACAGTGCCCG**	**ATTAAAGGCCGTAATCAGTAGCGAGCCACCCTCAGAGCCAGGAGGTTGAGGCAGGTAACAGTGCCCG**	**67**	**ATTAAAGGCCGTAATCAGTAG**	**51**	**CGGGCACTGTTACCTGCCTC**	**64**	**49**
46	**t7s4e**	**GCCGCCAGCATTGACACCACCCTC**	**t7s6e**	**AGAGCCGCACCATCGATAGCAGCATGAATTAT**	**GCCGCCAGCATTGACACCACCCTCAGAGCCGCACCATCGATAGCAGCATGAATTAT**	**56**	**GCCGCCAGCATTGACACCAC**	**65**	**ATAATTCATGCTGCTATCGATG**	**51**	**48**
47	**t-6s5c**	**GTTTGAAATTCAAATATATTTTAG**	**t-7s4e-t8s25c-2T**	**TTAATTTCATCTTAGACTTTACAA**	**GTTTGAAATTCAAATATATTTTAGTTAATTTCATCTTAGACTTTACAA**	**48**	**GTTTGAAATTCAAATATATTTTAG**	**42**	**TTGTAAAGTCTAAGATGAAA**	**44**	**41**
48	**t1s4i**	**AGCGTCATGTCTCTGAATTTACCGACTACCTT**	**t-1s4i**	**TTTAACCTATCATAGGTCTGAGAGTTCCAGTA**	**AGCGTCATGTCTCTGAATTTACCGACTACCTTTTTAACCTATCATAGGTCTGAGAGTTCCAGTA**	**64**	**AGCGTCATGTCTCTGAATTT**	**52**	**TACTGGAACTCTCAGACCTA**	**52**	**49**
49	**t-7s14e-t8s5c-2T**	**ATGACCCTGTAATACTTCAGAGCA**	**t7s4e**	**GCCGCCAGCATTGACACCACCCTC**	**ATGACCCTGTAATACTTCAGAGCAGCCGCCAGCATTGACACCACCCTC**	**48**	**TTTAATTGTATTTCCACCAGAG**	**49**	**GAGGGTGGTGTCAATGCTGG**	**61**	**46**
50	**t-4s5f**	**GTTAAATACAATCGCAAGACAAAGCCTTGAAA**	**t-4s3g**	**ACATAGCGCTGTAAATCGTCGCTATTCATTTCAATTACCT**	**GTTAAATACAATCGCAAGACAAAGCCTTGAAAACATAGCGCTGTAAATCGTCGCTATTCATTTCAATTACCT**	**72**	**GTTAAATACAATCGCAAGACAAAG**	**51**	**AGGTAATTGAAATGAATAGCGAC**	**51**	**49**

Nick #	Staple 1	Sequence	Staple 2	Sequence	Total sequence	Total length	Primer 1	T_m (°C)	Primer 2	T_m (°C)	T_{anneal} (°C)
51	t-4s1g	GAGCAAAAGAAGATGAGTGAATAACCTTGCTTATAGCTTA	t-3s4e	GATTAAGAAATGCTGATGCAAATCAGAATAAA	GAGCAAAAGAAGATGAGTGAATAACCTTGCTTATAGCTTAGATTAAGAAATGCTGATGCAAATCAGAATAAA	72	GAGCAAAAGAAGATGAGTGA	51	TTTATTCTGATTTGCATCAGC	50	49
52	t-2s5f	ACTAGAAATATATAACTATATGTACGCTGAGA	t-2s1g	AAAACAAAATTAATTAAATGGAAACAGTACATTAGTGAAT	ACTAGAAATATATAACTATATGTACGCTGAGAAAAACAAAATTAATTAAATGGAAACAGTACATTAGTGAAT	72	AGAGTCAAAAATCAATATATGTGAT	48	ATTCACTAATGTACTGTTTCC	48	46
53	t-2s1g	AAAACAAAATTAATTAAATGGAAACAGTACATTAGTGAAT	t-1s4e	TTATCAAACCGGCTTAGGTTGGGTAAGCCTGT	AAAACAAAATTAATTAAATGGAAACAGTACATTAGTGAATTTATCAAACCGGCTTAGGTTGGGTAAGCCTGT	72	AAAACAAAATTAATTAAATGGAAAC	45	ACAGGCTTACCCAACCTAAG	56	41
54	t-1s4i	TTTAACCTATCATAGGTCTGAGAGTTCCAGTA	t1s4i	AGCGTCATGTCTCTGAATTTACCGACTACCTT	TTTAACCTATCATAGGTCTGAGAGTTCCAGTAAGCGTCATGTCTCTGAATTTACCGACTACCTT	64	TTTAACCTATCATAGGTCTGAG	49	AAGGTAGTCGGTAAATTCAG	50	46
55	t2s5f	CCGGAACCCAGAATGGAAAGCGCAACATGGCT	t2s3g	TTTGATGATTAAGAGGCTGAGACTTGCTCAGTACCAGGCG	CCGGAACCCAGAATGGAAAGCGCAACATGGCTTTTGATGATTAAGAGGCTGAGACTTGCTCAGTACCAGGCG	72	CCGGAACCCAGAATGGAAAG	58	CGCCTGGTACTGAGCAAGTC	61	54
56	t2s1g	GATAAGTGCCGTCGAGCTGAAACATGAAAGTATACAGGAG	t3s4e	TGTACTGGAAATCCTCATTAAAGCAGAGCCAC	GATAAGTGCCGTCGAGCTGAAACATGAAAGTATACAGGAGTGTACTGGAAATCCTCATTAAAGCAGAGCCAC	72	GATAAGTGCCGTCGAGCTGA	59	GTGGCTCTGCTTTAATGAGG	55	54
57	t4s5f	CTCAGAGCATATTCACAAACAAATTAATAAGT	t4s3g	TTTAACGGTTCGGAACCTATTATTAGGGTTGATATAAGTA	CTCAGAGCATATTCACAAACAAATTAATAAGTTTTAACGGTTCGGAACCTATTATTAGGGTTGATATAAGTA	72	CTCAGAGCATATTCACAAAC	50	TACTTATATCAACCCTAATAATAGG	47	46
58	t4s1g	TAGCCCGGAATAGGTGAATGCCCCCTGCCTATGGTCAGTG	t5s4e	CCTTGAGTCAGACGATTGGCCTTGCGCCACCC	TAGCCCGGAATAGGTGAATGCCCCCTGCCTATGGTCAGTGCCTTGAGTCAGACGATTGGCCTTGCGCCACCC	72	TAGCCCGGAATAGGTGAATG	56	GGGTGGCGCAAGGCCAATCG	68	54
59	t1s2i	CGGGGTTTCCTCAAGAGAAGGATTTGAATTA	t-1s2i	CCTTTTTTCATTTAACAATTTCATAGGATTAG	CGGGGTTTCCTCAAGAGAAGGATTTTGAATTACCTTTTTTCATTTAACAATTTCATAGGATTAG	64	CGGGGTTTCCTCAAGAGAAG	57	CTAATCCTATGAAATTGTTAAATGA	47	46
60	t-4s3g	ACATAGCGCTGTAAATCGTCGCTATTCATTTCAATTACCT	t-4s1g	GAGCAAAAGAAGATGAGTGAATAACCTTGCTTATAGCTTA	ACATAGCGCTGTAAATCGTCGCTATTCATTTCAATTACCTGAGCAAAAGAAGATGAGTGAATAACCTTGCTTATAGCTTA	80	ACATAGCGCTGTAAATCGTC	54	TAAGCTATAAGCAAGGTTATTCA	50	49

Nick #	Staple 1	Sequence	Staple 2	Sequence	Total sequence	Total length	Primer 1	T_m (°C)	Primer 2	T_m (°C)	T_{anneal} (°C)
61	t-2s3g	AGAGTCAAAAATCAAT ATATGTGATGAAACAA ACATCAAG	t-2s1g	AAAACAAAATTAATT AAATGGAAACAGTAC ATTAGTGAAT	AGAGTCAAAAATCAATATATGTGAT GAAACAAACATCAAGAAAACAAA ATTAATTAAATGGAAACAGTACATT AGTGAAT	80	AGAGTCAAAA ATCAATATATGT GAT	48	ATTCACTAAT GTACTGTTT CC	48	46
62	t-1s2i	CCTTTTTTCATTTAACA ATTTCATAGGATTAG	t1s2i	CGGGGTTTCCTCAAG AGAAGGATTTTGAAT TA	CCTTTTTTCATTTAACAATTTCATAG GATTAGCGGGGTTTCCTCAAGAGA AGGATTTTGAATTA	64	CCTTTTTTCATT TAACAATTTCA TA	46	TAATTCAAA ATCCTTCTCT TGA	47	46
63	t2s3g	TTTGATGATTAAGAGG CTGAGACTTGCTCAGT ACCAGGCG	t2s1g	GATAAGTGCCGTCGA GCTGAAACATGAAAG TATACAGGAG	TTTGATGATTAAGAGGCTGAGACTT GCTCAGTACCAGGCGGATAAGTGC CGTCGAGCTGAAACATGAAAGTAT ACAGGAG	80	TTTGATGATTA AGAGGCTGA	49	CTCCTGTATA CTTTCATGTT	47	46
64	t4s3g	TTTAACGGTTCGGAAC CTATTATTAGGGTTGAT ATAAGTA	t4s1g	TAGCCCGGAATAGGT GAATGCCCCCTGCCT ATGGTCAGTG	TTTAACGGTTCGGAACCTATTATTA GGGTTGATATAAGTATAGCCCGGAA TAGGTGAATGCCCCCTGCCTATGGT CAGTG	80	TTTAACGGTTC GGAACCTAT	52	CACTGACCA TAGGCAGGG GG	63	49

Table S3. *Oligonucleotides for calibrations.*

Experiment	Sequence	Length (nt)	Forward primer	T_m (°C)	Reverse primer	T_m (°C)	T_{anneal} (°C)
Absolute quantification of DNA nanostructures' scaffold strand	CTGTTGCAGGCGGTGTTAATACTGA CCGCCTCACCTCTGTTTTATCTTCT GCTGGTGGTTCGTT	64	AACGAACCACCAGCAGAAGA	58	CTGTTGCAGGCGGTGTTAAT	58	52
Absolute quantification of ligated staple strands	ACGACAATAAATCCCGACTTGCGG GAGATCCTGAATCTTACCAACGCTA ACGAGCGTCTGGCGTTTTAGCGAA CCCAACATGT	83	ACGACAATAAATCCCGACTT	52	ACATGTTGGGTTCGCTAAAA	53	49
